# Sex differences in coronary angiographic findings in patients with stable chest pain: analysis of data from the KoRean wOmen’S chest pain rEgistry (KoROSE)

**DOI:** 10.1186/s13293-021-00411-1

**Published:** 2022-01-03

**Authors:** Hack-Lyoung Kim, Hyun-Jin Kim, Mina Kim, Sang Min Park, Hyun Ju Yoon, Young Sup Byun, Seong Mi Park, Mi Seung Shin, Kyung-Soon Hong, Myung-A Kim

**Affiliations:** 1 Division of Cardiology, Department of Internal Medicine, Seoul National University College of Medicine, Boramae Medical Center, Seoul, 07061 Korea; 2 Division of Cardiology, Department of Internal Medicine, Hanyang University Guri Hospital, Guri, Korea; 3 Division of Cardiology, Department of Internal Medicine, Korea University Anam Hospital, Seoul, Korea; 4 Division of Cardiology, Department of Internal Medicine, Nowon Eulji Medical Center, Eulji University, Seoul, Korea; 5 Division of Cardiology, Department of Internal Medicine, Chonnam National University Hospital, Gwangju, Korea; 6 Division of Cardiology, Department of Internal Medicine, Inje University Sanggye Paik Hospital, Seoul, Korea; 7 Division of Cardiology, Department of Internal Medicine, Gachon Medical School Gil Medical Center, Incheon, Korea; 8 Division of Cardiology, Department of Internal Medicine, Hallym University Medical Center, Chuncheon, Korea

**Keywords:** Coronary angiography, Coronary artery disease, Left main disease, Sex differences, Three-vessel disease

## Abstract

**Background:**

Focused evaluations on potential sex differences in the angiographic findings of the coronary arteries are scarce. This study was performed to compare the angiographic extent and localization of coronary stenosis between men and women.

**Methods:**

A total of 2348 patients (mean age 62.5 years and 60% women) with stable chest pain undergoing invasive coronary angiography (CAG) were recruited from the database of the nation-wide chest pain registry. Obstructive coronary artery disease (CAD) was defined as ≥ 50% stenosis of the left main coronary artery and/or ≥ 70% stenosis of any other epicardial coronary arteries.

**Results:**

Although women were older than men (64.4 ± 10.3 vs. 59.5 ± 11.4 years, *P* < 0.001), men had worse risk profiles including high blood pressure, more frequent smoking and elevated triglyceride and C-reactive protein. The prevalence of obstructive CAD was significantly higher in men than in women (37.0% vs. 28.4%, *P* < 0.001). Men had a higher prevalence of LM disease (10.3% vs. 3.5%, *P* < 0.001) and three-vessel disease (16.1% vs. 9.5%, *P* = 0.007) compared to women. In multiple binary logistic regression analysis, the risk of men having LM disease or three-vessel disease was 7.4 (95% confidence interval 3.48–15.97; *P* < 0.001) and 2.7 (95% confidence interval 1.57–4.64; *P* < 0.001) times that of women, respectively, even after controlling for potential confounders.

**Conclusions:**

In patients with chest pain undergoing invasive CAG, men had higher obstructive CAD prevalence and more high-risk angiographic findings such as LM disease or three-vessel disease.

**Supplementary Information:**

The online version contains supplementary material available at 10.1186/s13293-021-00411-1.

## Abstract (article highlights)

- Focused evaluation on sex differences in the angiographic findings of the coronary arteries is limited.
- We evaluated sex differences in the angiographic extent and localization of coronary stenosis in 2348 patients.
- Men had higher prevalence of obstructive coronary artery disease and more high-risk angiographic findings such as left main disease or three-vessel disease.
- This sex difference could be applied in the clinical evaluation and management of patients with suspected coronary artery disease.

**Supplementary Information:**

The online version contains supplementary material available at 10.1186/s13293-021-00411-1.

## Introduction

Coronary artery disease (CAD) is a leading cause of morbidity and mortality worldwide. With improvement in diagnostic and therapeutic tools, the prognosis of patients with CAD has been much improved. However, the prevalence of CAD is still high, and the complications associated with CAD are the number one cause of human death [1–3] Therefore, in order to improve patients’ prognosis and reduce the enormous medical cost, it is important to find CAD patients earlier and perform customized treatment. For rapid CAD diagnosis and effective treatment, understanding pathophysiology underlying in CAD development should be the basis. Human efforts to understand sex differences in the cardiovascular field, and to apply them in clinical practice have continued [4–6]. In CAD, sex differences in several points such as clinical presentation and prognosis are relatively well evaluated. However, little is known regarding potential sex differences in the angiographic findings of coronary arteries. Since invasive coronary angiography (CAG) is the reference standard for CAD diagnosis, understanding sex difference in invasive CAG findings is valuable for the management of patients with CAD. Therefore, this study was performed to compare the extent and localization of coronary stenosis on invasive CAG between men and women.

## Materials and methods

### Study patients

We analyzed data from the nation-wide prospective registry database, the KoRean wOmen’S chest pain rEgistry (KoROSE), which was constructed to investigate clinical characteristics and outcomes of Korean women with suspected CAD in a stable state. For comparison, men were also registered in the registry. Many research articles using this registry data have already been published [7–9]. Collection of this registry data began in February 2011, and patient registration is still ongoing. Currently, 22 cardiovascular centers in Korea are participating in this registry. The patients enrolled were Korean adult men and women over the age of 20 years who complained of chest pain and underwent invasive CAG because of suspected CAD. Because study enrollment was based on relatively stable patients who visited the outpatient clinic, patients with acute coronary syndrome were excluded. In most cases, tests such as treadmill exercise test, coronary computed tomography angiography, single-photon emission computed tomography, and dobutamine stress echocardiography were performed according to the patient’s renal function and functional capacity. Invasive CAG was performed according to these results of non-invasive tests. After invasive CAG, attending physician explained the study protocol and enrolled patients who agreed to participate in the registry. The Institutional Review Board of Boramae Medical Center (Seoul, South Korea) approved registry registration, and the use of the registered data for research purposes. All patients were given written consent for registry registration.

### Data collection

Clinical data were obtained at the time of admission for invasive CAG. Body mass index was the body weight (kg) divided by the height squared (m^2^). Body mass index ≥ 25 kg/m^2^ was considered obese [10]. Waist circumference was measured with a tape measure. A tape measure was placed in the middle of the lowest position of the ribs and the highest position of the pelvis during expiration. Systolic/diastolic blood pressure and heart rate were measured by a trained nurse using an automatic oscillometric device. Hypertension was defined on the basis of (1) previous diagnosis of hypertension by a physician; (2) current anti-hypertensive medications, or (3) systolic/diastolic blood pressure ≥ 140/90 mmHg in repeated measurements. Diabetes mellitus was defined on the basis of (1) previous diagnosis of diabetes mellitus by a physician; (2) current anti-diabetic medications, or (3) fasting blood glucose level ≥ 126 mg/dL in repeated tests. Dyslipidemia was defined on the basis of (1) previous diagnosis of dyslipidemia by a physician; (2) current anti-dyslipidemic medications, or (3) low-density lipoprotein cholesterol ≥ 160 mg/dL. A person who smoked regularly within the last 12 months was defined as a smoker. After overnight fasting, blood levels of the following laboratory parameters were obtained: white blood cell count, hemoglobin, creatinine, glucose, glycated hemoglobin, total cholesterol, low-density lipoprotein cholesterol, high-density lipoprotein cholesterol (HDL-C), triglyceride, and C-reactive protein. Estimated glomerular filtration rate was calculated using the Modification of Diet in Renal Disease (MDRD) Study equation. Information on concomitant cardiovascular medications including antiplatelets, calcium channel blocker, beta-blocker, renin–angiotensin system blocker, and statin was also obtained.

### Invasive CAG

Invasive CAG was performed using a radial or femoral artery in accordance with current guidelines [11, 12]. All management strategies for CAD, including coronary revascularization, were chosen at the discretion of the attending physician. An obstructive CAD was defined as any ≥ 50% stenosis of the left main coronary artery, ≥ 70% stenosis of any other epicardial coronary arteries, or both. The extent of CAD was classified as one‐, two‐, or three‐vessel disease. Significant left main stenosis (≥ 50%) was considered as two-vessel diseases. The coronary artery was divided into 17 segments, and we obtained information on the maximum stenosis of each segment [13, 14]. Left main disease or three-vessel disease were considered as a high-risk finding.

### Statistical analysis

Continuous variables are expressed as mean ± standard deviation, and categorical variables are expressed as *n* (%). Student’s *t* test was used to compare continuous variables and the Chi-square test was used to compare categorical variables between two groups. Binary logistic regression analyses were performed to investigate independent associations between sex and angiographic findings. During multivariable analyses the following potential confounders were controlled: age, body mass index, hypertension, diabetes mellitus, dyslipidemia, smoking and renal function. A *P* value of < 0.05 was considered statistically significant. All statistical analyses were conducted using SPSS version 21.0 (IBM Crop., Armonk, NY, USA).

## Results

### Clinical characteristics of the study patients

Nine hundred and forty-one men and 1407 women (59.9%) were analyzed in this study. Comparisons of clinical characteristics between men and women are demonstrated in Table 1. Women were older than men (64.4 ± 10.3 vs. 59.5 ± 11.4 years, *P* < 0.001). Mean body mass index was similar between men and women, but waist circumference was greater in men than in women. Both systolic and diastolic blood pressures were higher in men than in women. Among traditional cardiovascular risk factors, the proportions of smokers and obesity were higher in men than in women. In laboratory findings, women had lower blood hemoglobin, triglycerides and C-reactive protein levels as well as higher total cholesterol and high-density lipoprotein cholesterol levels than men. Among cardiovascular medications, beta-blockers and statin were more frequently prescribed in women than in men.

**Table 1:** Clinical characteristics of study patients according to sex

Characteristic	Men (*n* = 941)	Women (*n* = 1407)	*P* value
Age, years	59.5 ± 11.4	64.4 ± 10.3	< 0.001
Body mass index, kg/m^2^	25.1 ± 3.1	25.1 ± 3.7	0.905
Body mass index ≥ 25 kg/m^2^, %	51.5	46.2	0.014
Waist circumference (WC), cm	86.7 ± 9.5	82.7 ± 10.0	< 0.001
WC ≥ 90 cm for men and ≥ 85 cm for women, %	35.9	38.6	0.348
Systolic blood pressure, mmHg	128 ± 17	126 ± 18	0.025
Diastolic blood pressure, mmHg	79.0 ± 11.6	75.4 ± 11.6	< 0.001
Heart rate, per minute	74.7 ± 13.6	74.5 ± 12.7	0.782
Cardiovascular risk factors, %			
Hypertension	53.6	56.0	0.255
Diabetes mellitus	27.0	23.9	0.110
Dyslipidemia	25.2	26.7	0.491
Current smoking	35.0	4.9	< 0.001
Obesity (body mass index ≥ 25 kg/m^2^)	51.5	46.2	0.014
Laboratory findings			
White blood cell count, per microliter	6951 ± 2118	6811 ± 2647	0.197
Hemoglobin, g/dL	14.3 ± 1.6	12.8 ± 1.4	< 0.001
Glomerular filtration rate, mL/min/1.73 m^2^	81.5 ± 21.3	83.3 ± 29.1	0.102
Fasting glucose, mg/dL	119 ± 44	118 ± 46	0.755
Glycated hemoglobin, %	6.25 ± 1.25	6.17 ± 1.12	0.356
Total cholesterol, mg/dL	164 ± 41	172 ± 44	< 0.001
Low-density lipoprotein cholesterol, mg/dL	101 ± 36	103 ± 36	0.156
High-density lipoprotein cholesterol, mg/dL	43.6 ± 11.6	49.3 ± 13.2	< 0.001
Triglycerides, mg/dL	154 ± 111	127 ± 87	< 0.001
C-reactive protein, mg/dL	2.12 ± 8.83	0.97 ± 3.24	0.007
Concomitant medications, %			
Antiplatelets	48.8	51.2	0.287
Calcium channel blocker	33.7	34.0	0.896
Beta-blocker	23.1	29.2	0.003
Renin–angiotensin system blocker	39.3	39.6	0.874
Statin	51.4	56.6	0.022

About one-third of patients (31.8%) had obstructive CAD. Comparisons of clinical characteristics between patients with and without obstructive CAD in men and women are shown in Table 2. Patients with obstructive CAD were older in both men (62.6 ± 10.2 vs. 57.0 ± 11.6 years, *P* < 0.001) and women (68.1 ± 9.1 vs. 62.4 ± 10.3 years, *P* < 0.001). Patients with obstructive CAD had more cardiovascular risk factors such as hypertension, diabetes and smoking than those without in both sexes. Laboratory findings also showed worse profiles including higher levels of white blood cell count, fasting glucose and low-density lipoprotein cholesterol as well as lower levels of hemoglobin, glomerular filtration rate, and high-density lipoprotein cholesterol in patients with obstructive CAD than in those without in both men and women. Antiplatelets, beta-blocker, renin–angiotensin system blocker and statin were more frequently prescribed to patients with obstructive CAD than those without in both sexes.

**Table 2:** Clinical characteristics of study patients according to the presence of obstructive CAD and sex

Characteristic	Men (*n* = 941)			Women (*n* = 1407)		
	Obstructive CAD (−) (*n* = 517)	Obstructive CAD (+) (*n* = 424)	*P* value	Obstructive CAD (−) (*n* = 907)	Obstructive CAD (+) (*n* = 500)	*P* value
Age, years	57.0 ± 11.6	62.6 ± 10.2	< 0.001	62.4 ± 10.3	68.1 ± 9.1	< 0.001
BMI, kg/m^2^	25.0 ± 3.1	25.2 ± 3.1	0.536	25.1 ± 3.7	25.0 ± 3.7	0.389
Waist circumference, cm	86.0 ± 9.0	87.6 ± 10.1	0.082	82.0 ± 10.1	83.9 ± 9.6	0.016
Systolic blood pressure, mmHg	126 ± 16	130 ± 18	0.005	124 ± 17	130 ± 20	< 0.001
Diastolic blood pressure, mmHg	79.7 ± 11.5	78.2 ± 11.7	0.088	75.1 ± 11.3	76.1 ± 12.2	0.188
Heart rate, per minute	75.1 ± 13.9	74.3 ± 13.2	0.426	74.2 ± 12.2	74.9 ± 13.5	0.418
Cardiovascular risk factors, %						
Hypertension	47.4	61.1	< 0.001	49.3	67.7	< 0.001
Diabetes mellitus	19.6	36.2	< 0.001	17.8	34.6	< 0.001
Dyslipidemia	26.0	24.5	0.653	27.3	25.6	0.526
Current smoking	31.5	39.1	0.021	4.9	4.8	0.972
Obesity (BMI ≥ 25 kg/m^2^)	51.1	52.0	0.807	46.0	46.4	0.889
Laboratory findings						
WBC, per microliter	6764 ± 2118	7175 ± 2098	0.005	6471 ± 2237	7405 ± 3156	< 0.001
Hemoglobin, g/dL	14.5 ± 1.5	14.0 ± 1.6	< 0.001	12.9 ± 1.3	12.5 ± 1.6	< 0.001
GFR, mL/min/1.73 m^2^	82.1 ± 19.4	80.8 ± 23.4	0.356	84.1 ± 27.5	81.9 ± 31.6	0.204
Fasting glucose, mg/dL	113 ± 36	127 ± 52	< 0.001	112 ± 40	129 ± 53	< 0.001
Glycated hemoglobin, %	5.97 ± 1.05	6.58 ± 1.38	< 0.001	6.04 ± 0.99	6.39 ± 1.26	< 0.001
Total cholesterol, mg/dL	165 ± 40	162 ± 43	0.218	171 ± 43	173 ± 46	0.491
LDL cholesterol, mg/dL	102 ± 34	100 ± 38	0.435	101 ± 35	107 ± 37	0.018
HDL cholesterol, mg/dL	45.2 ± 12.2	41.6 ± 10.6	< 0.001	51.1 ± 13.9	45.8 ± 11.1	< 0.001
Triglyceride, mg/dL	155 ± 108	152 ± 116	0.776	123 ± 75	133 ± 105	0.079
C-reactive protein, mg/dL	2.74 ± 11.5	1.39 ± 3.56	0.101	0.79 ± 2.47	1.28 ± 4.22	0.081
Concomitant medications, %						
Antiplatelet	36.9	63.8	< 0.001	43.1	65.9	< 0.001
Calcium channel blocker	29.1	39.6	0.002	33.5	34.9	0.632
Beta-blocker	15.9	32.3	< 0.001	23.5	39.8	< 0.001
RAS blocker	33.0	47.3	< 0.001	34.5	49.0	< 0.001
Statin	42.6	62.7	< 0.001	52.4	64.3	< 0.001

*CAD* coronary artery disease; *BMI* body mass index; *WBC* white blood cell; *GFR* glomerular filtration rate; *LDL* low-density lipoprotein; *HDL* high-density lipoprotein; *RAS* renin–angiotensin system

### Sex differences in angiographic findings

**Fig. 1:**
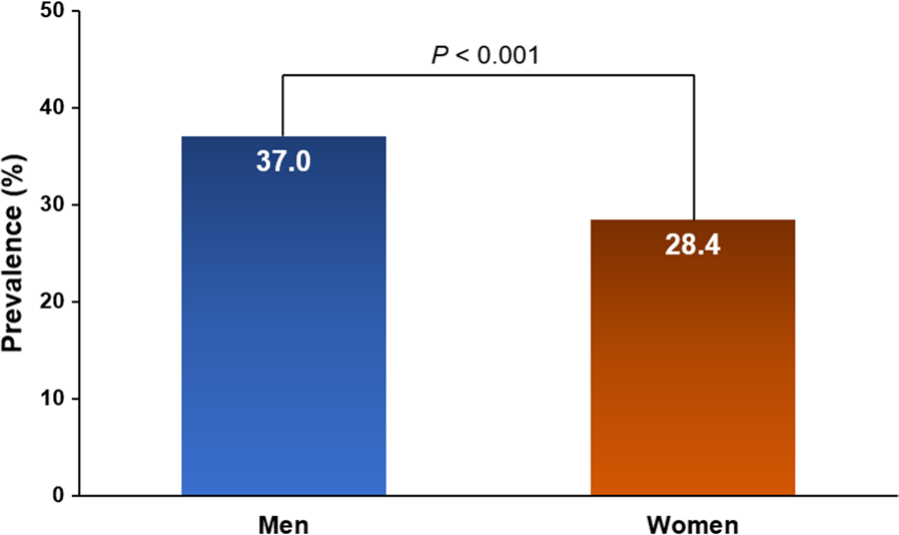
Prevalence of obstructive coronary artery disease in men and women

**Fig. 2:**
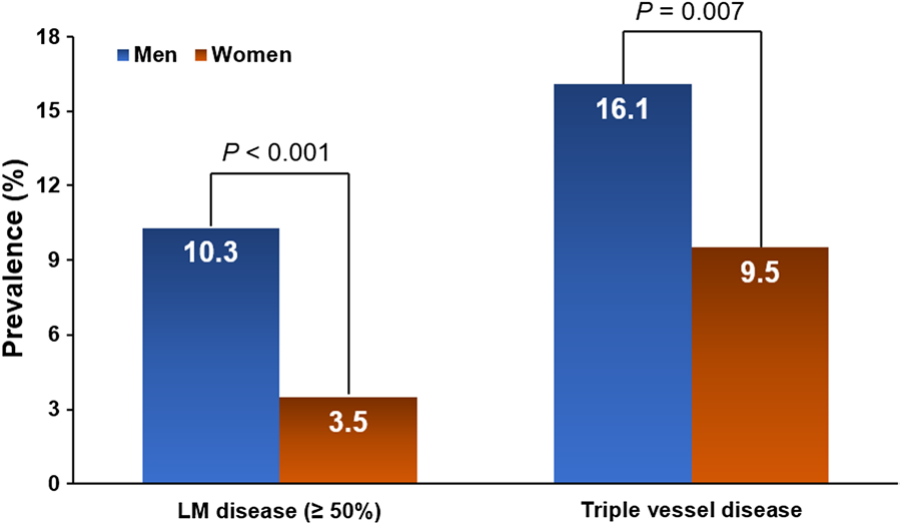
Prevalence of LM disease and triple-vessel disease in men and women. *LM* left main

Angiographic findings of men and women in the total study population are demonstrated in Table 3. The prevalence of obstructive CAD was significantly higher in men than in women (37.0% vs. 28.4%, *P* < 0.001) (Fig. 1). Two- or three-vessel disease or LM disease was more prevalent in men than in women (16.0% vs. 11.2%, *P* ≤ 0.001). In addition to the three major epicardial coronary arteries, significant stenosis of the branch arteries was also prevalent in men, compared to women. LM disease with proximal LAD significant stenosis or LM with proximal significant stenosis of at least one of three major epicardial coronary arteries were more frequently observed in men than in women. Even when we considered only patients with obstructive CAD, men had more three-vessel disease or LM disease than in women (*P* < 0.05 for each) (Fig. 2). Significant stenosis of the RCA and branched coronary arteries were more prevalent in men than in women (Table 4). Being a man itself was an independent factor predicting obstructive CAD (OR [odds ratio] 1.48; 95% CI [confidence interval] 1.17–1.86; *P* = 0.001), LM disease (OR 7.46; 95% CI 3.48–15.98; *P* < 0.001), LM disease with proximal LAD significant stenosis (OR 1.51; 95% CI 1.16–1.98; *P* = 0.002), and three-vessel disease (OR 2.70; 95% CI 1.57–4.64; *P* < 0.001), even though various clinically important covariates were corrected (Table 5). Besides male sex, old age was associated with LM disease, and old age, hypertension and diabetes mellitus were associated with three-vessel disease even after controlling for potential confounders (Additional file 1: Table S1).

**Table 3:** Angiographic findings according to sex in total population

Characteristic	Men (*n* = 941)	Women (*n* = 1407)	*P* value
Obstructive CAD (LM ≥ 50%, other ≥ 70%)	348 (37.0)	400 (28.4)	< 0.001
Insignificant	593 (63.0)	1007 (71.6)	< 0.001
One-vessel disease	197 (20.9)	242 (17.2)	
Two-vessel disease	95 (10.1)	120 (8.5)	
Three-vessel disease	56 (6.0)	38 (2.7)	
Two- or three-vessel disease	151 (16.0)	158 (11.2)	0.001
LM disease (≥ 50%)	36 (3.8)	14 (1.0)	< 0.001
LAD stenosis			
Total LAD ≥ 70%	233 (24.8)	277 (19.7)	0.003
Proximal LAD ≥ 70%	116 (12.3)	140 (10.0)	0.070
Mid-LAD ≥ 70%	101 (10.7)	132 (9.4)	0.283
Distal LAD ≥ 70%	19 (2.0)	21 (1.5)	0.334
LCX stenosis			
Total LCX ≥ 70%	140 (14.9)	161 (11.4)	0.015
Proximal LCX ≥ 70%	55 (5.8)	59 (4.2)	0.068
Distal LCX ≥ 70%	69 (7.3)	94 (6.7)	0.543
RCA stenosis			
Total RCA ≥ 70%	153 (16.3)	147 (10.4)	< 0.001
Proximal RCA ≥ 70%	51 (5.4)	52 (3.7)	0.046
Mid-RCA ≥ 70%	51 (5.4)	64 (4.5)	0.338
Distal RCA ≥ 70%	50 (5.3)	43 (3.1)	0.006
Branched artery stenosis			
Diagonal ≥ 70%	45 (4.8)	25 (1.8)	< 0.001
OM ≥ 70%	25 (2.7)	12 (0.9)	0.001
PDA or PL ≥ 70%	28 (3.0)	13 (0.9)	< 0.001
LM disease (≥ 50%) and pLAD stenosis (≥ 70%)	244 (25.9)	283 (20.1)	0.001
LM disease (≥ 50%) and proximal stenosis (≥ 70%)	348 (37.0)	400 (28.4)	< 0.001

Numbers are expressed as n (%)

*CAD* coronary artery disease; *LM* left main; *LAD* left anterior descending artery; *LCX* left circumflex artery; *RCA* right coronary artery; *OM* obtuse marginal artery; *PDA* posterior descending artery; *PL* posterior longitudinal artery

**Table 4:** Angiographic findings according to sex in patients with obstructive CAD

Characteristic	Men (*n* = 348)	Women (*n* = 400)	*P* value
One-vessel disease	197 (56.6)	242 (60.5)	0.025
Two-vessel disease	95 (27.3)	120 (30.3)	
Three-vessel disease	56 (16.1)	38 (9.5)	
Two- or three-vessel disease	151 (43.4)	309 (41.3)	0.281
Three-vessel disease	56 (16.1)	38 (9.5)	0.007
LM disease (≥ 50%)	36 (10.3)	14 (3.5)	< 0.001
LAD stenosis			
Total LAD ≥ 70%	233 (67.0)	277 (69.2)	0.501
Proximal LAD ≥ 70%	116 (33.3)	140 (35.0)	0.632
Mid-LAD ≥ 70%	101 (29.0)	132 (33.0)	0.241
Distal LAD ≥ 70%	19 (5.5)	21 (5.2)	0.899
LCX stenosis			
Total LCX ≥ 70%	140 (40.2)	161 (40.2)	0.996
Proximal LCX ≥ 70%	55 (15.8)	59 (14.8)	0.689
Distal LCX ≥ 70%	69 (19.8)	94 (23.5)	0.225
RCA stenosis			
Total RCA ≥ 70%	153 (44.0)	147 (36.8)	0.045
Proximal RCA ≥ 70%	51 (14.7)	52 (13.0)	0.512
Mid-RCA ≥ 70%	51 (14.7)	64 (16.0)	0.611
Distal RCA ≥ 70%	50 (14.4)	43 (10.8)	0.135
Branched artery stenosis			
Diagonal ≥ 70%	45 (12.9)	25 (6.2)	0.002
OM ≥ 70%	25 (7.2)	13 (3.0)	0.008
PDA or PL ≥ 70%	28 (8.0)	13 (3.2)	0.004
LM disease (≥ 50%) and pLAD stenosis (≥ 70%)	244 (70.1)	283 (70.8)	0.849
LM disease (≥ 50%) and proximal stenosis (≥ 70%)	348 (100)	400 (100)	1.000

Numbers are expressed as n (%)

*CAD* coronary artery disease; *LM* left main; *LAD* left anterior descending artery; *LCX* left circumflex artery; *RCA* right coronary artery; *OM* obtuse marginal artery; *PDA* posterior descending artery; *PL* posterior longitudinal artery

**Table 5:** Association between sex and CAD

Variable	OR (95% CI)	*P* value
Obstructive CAD		
Men (vs. women)	1.50 (1.14–1.96)	0.003
LM disease (≥ 50%)		
Men (vs. women)	5.84 (2.55–13.3)	< 0.001
LM disease (≥ 50%) and pLAD stenosis (≥ 70%)		
Men (vs. women)	1.56 (1.18–2.05)	0.001
Three-vessel disease		
Men (vs. women)	2.51 (1.37–4.60)	0.003

Age, body mass index, hypertension, diabetes mellitus, dyslipidemia, smoking, renal function, and the use of antiplatelet, beta-blocker, renin–angiotensin system blocker and statin were adjusted

*CAD* coronary artery disease; *OR* odds ratio; *CI* confidence interval; *LM* left main; *LAD* left anterior descending artery

## Discussion

Using a nation-wide registry database, we attempted to find out the sex differences of invasive CAG findings in patients who had chest pain in a stable state. Our results showed several important findings: (1) despite younger age, men more frequently had risk factors for cardiovascular disease than women, which resulted in a higher obstructive CAD prevalence in men; (2) men more frequently had LM disease or three-vessel disease than women, and (3) these sex differences persisted even after controlling for important clinical covariates.

### Previous similar studies

The summary of previous studies investigating sex differences in CAG findings is demonstrated in Table 6. Numerous studies have reported that angiographically documented CAD is more severe in men than in women [15–22]. These findings are concordant with ours showing that compared to women, men had more burden of obstructive CAD. However, our study has several differences and strengths compared to the previous studies. Most of the existing studies were conducted in the Western countries. If we consider ethnic differences in cardiovascular disease [23], our study of Koreans is valuable. Our study provides an additional result that in Asians like Westerners, men more frequently have CAD than women. Considering very low proportion of women in previous studies, higher proportion of women was another strength of this study. In addition, the primary research goal in most studies was to determine if there were sex differences in subsequent management and clinical outcomes following CAG. Therefore, only sex differences in CAG findings were demonstrated briefly, and more specific analysis on lesion location were not shown in most studies. Only one study focused primarily on sex differences in angiographic findings, and showed specific CAD locations [15]. Moreover, only a few studies have performed multivariable analysis to demonstrate whether sex is an independent factor associated with CAD severity [18, 22]. In our study, the primary aim was to determine the differences in CAG findings between men and women. In addition, we analyzed the detailed lesion location of CAD, and also performed multivariable analysis to evaluate the effect of sex on CAD severity after adjustment for confounding factors. Although not the majority opinion, some other studies have shown that there is no sex difference in the extent and localization of coronary angiographic lesions [24–27]. Further studies are needed to reach a firmer conclusion on sex difference in the severity and extent of angiographic CAD.

**Table 6:** Summary of previous studies on sex differences in CAG findings

Source	Area or country	Number of study subjects	Population	Female (%)	Primary research goal	Findings on sex difference of CAG findings
Giannoglou et al. [15]	Greece	14,090	Suspected CAD	12.9	To investigate sex differences of angiographic findings	Significant stenosis (≥ 50%) were more common in men (86% vs. 64%; *P* < 0.001) than in women
Gudnadottir et al. [16]	Sweden	106,881	Acute coronary syndrome	31.9	To investigate gender disparities in revascularization and clinical outcomes	Both left main stem stenosis and three-vessel disease were more common in men than in women (30.4% vs. 20.9%; *P* < 0.001)
Ouellette et al. [17]	USA	925	Suspected CAD	44.4	To investigate clinical characteristics and outcome of normal or near-normal coronary artery stenosis	More women than men (53.5% vs. 37.2%; *P* < 0.001) had normal or near-normal coronary arteries or non-obstructive CAD
Patel et al. [18]	USA	397,954	Suspected CAD	47.3	To investigate the diagnostic yield of invasive CAG	Male sex was an independent predictor for obstructive CAD (adjust OR 2.70; 95% CI 2.64–2.76)
Ritsinger et al. [19]	Sweden	2776	Type 1 diabetes undergoing CAG	42.0	To investigate sex aspects on CAD extent and prognosis in patients with type 1 diabetes	Three-vessel disease or left main disease were more common in men than in women (40.4% vs. 34.5%; *P* = 0.002)
Chiha et al. [20]	Australia	994	Suspected CAD	28.0	To investigate sex difference in CAG findings	Compared to men, women had lower mean extent scores (19.6 vs. 36.8; *P* < 0.0001) and lower vessel scores (0.7 vs. 1.3; *P* < 0.0001)
Bell et al. [21]	USA	22,795	Suspected CAD	17.3	To investigate gender bias in the selection for revascularization	Three-vessel disease was more frequently observed in men compared to women (41% vs. 26%, *P* < 0.0001)
Tamis-Holland et al. [22]	USA	1775	Type 2 diabetes with CAD	30.0	To investigate gender differences in symptoms and extent of CAD	Number of significant lesions was higher (2.7 ± 1.8 vs. 2.3 ± 1.7; *P* < 0.001) and total occlusion were more common (42% vs. 29%; *P* < 0.001) in men than in women
Roeters van Lennep et al. [24]	Netherlands	1894	With documented CAD	19.4	To investigate gender-related differences in CAD extent and localization	There were no significant differences in the prevalence of three-vessel disease (31.8% vs. 29.4%) and left main disease (6.4% vs. 8.1%) between men and women (*P* = 0.839)
Leaf et al. [25]	USA	1187	Suspected CAD	21.6	To investigate sex difference in CAG findings	There were no significant differences in the prevalence of three-vessel disease (47.5% vs. 42.9%) and left main disease (8.6% vs. 8.6%) between men and women in patients with CAD (*P* > 0.05)
Kyridakidis et al. [26]	Greece	735	With documented CAD	26.1	To investigate sex difference in CAG findings	Three-vessel CAD less common in women than in men (16% vs. 35%; *P* < 0.001). Gensini index was significantly higher in men (59 vs. 52; *P* = 0.03). The location of coronary stenoses did not show differences between men and women
Kim et al. [27]	South Korea	1136	Patients who underwent fractional flow reserve measurement	26.4	To investigate the influence of sex on the relationship between total anatomical and physiologic disease burdens	There were no differences in angiographic diameter stenosis, SYNTAX score, or residual SYNTAX score between women and men

*CAG* coronary angiography; *CAD* coronary artery disease; *OR* odds ratio; *CI* confidence interval; *SYNTAX* synergy between percutaneous coronary intervention with Taxus and cardiac surgery

### Underlying mechanisms

In our study, higher blood pressure, greater proportions of smokers and obese patients, and worse lipid profiles could explain more significant and extensive CAD in men than in women. Although women were older than men, they less frequently had cardiovascular risk factors than men. Indeed, in addition to old age and male sex, traditional cardiovascular risk factors including hypertension and diabetes mellitus were significantly associated with the presence of three-vessel disease in our multivariable analysis. However, old age and male sex were only factors associated with LM disease, and the risk of male sex itself was higher than age. It can be assumed that male sex itself had a great influence on LM disease, and cardiovascular risk factors, which have a high prevalence in men, contributed to the development of three-vessel disease. Cardiovascular system protection by female sex hormone may be a commonly proposed reason for lower risk profiles in women [28]. In our study, women had higher HDL-C than men, and those with CAD had significantly lower HDL-C than those without CAD. This result suggests that HDL-C probably played an important role in CAD development and progression [29], and induced sex differences.

### Clinical implications

Our results of Asian patients did not differ significantly from the main finding of the existing Western studies indicating that men had a more severe angiographically documented CAD than women. We should always be aware of the high risk of male sex itself when treating patients. In other words, since women generally develop less coronary artery pathology compared to men at the same age, women with CAD represent a vulnerable subgroup and need special attention. In addition, given that men have more severe and extensive CAD, one can expect that women have less symptoms and better prognosis; however, previous studies have shown opposite findings [30]. As ischemia and ischemia-like symptoms are not solely related to the severity of atherosclerosis, we should not overlook the fact that coronary microvascular dysfunction or coronary spasm are a more common cause of stable ischemic heart disease in women [5, 6, 26].

### Study limitations

We acknowledge several limitations of the present study. First, coronary stenosis was visually evaluated in our study. If we had performed quantitative coronary analysis, more accurate data could have been obtained. Second, in our study, no intravascular evaluation or computed tomographic examination was performed, so we could not analyze differences between men and women in coronary plaque properties [4, 5, 31]. Third, the hemodynamical significance of CAD was not evaluated in our study. Lastly, since all subjects of our study were Koreans and patients with stable chest pain, it would be difficult to apply our results directly to other ethnic groups or patients with acute coronary syndrome.

## Perspectives and significance

Among Korean patients with chest pain in a stable state, men had more extensive and severe angiographic CAD compared to women even at younger ages. More critical CAD including LM disease and three-vessel disease were also more prevalent in men. We need to understand this observed sex differences, which could apply in the clinical evaluation and management of patients with suspected CAD. Specifically, since men are more likely to have severe CAD, it is desirable to recommend more active tests and intensified management to men with suspected CAD.

## Supplementary Information

**Additional file 1. Table S1.** Multiple binary logistic regression analyses showing independent predictors for LM disease and three vessel disease.

## Data Availability

The data that support the findings of this study are available from the corresponding author upon reasonable request.
