# Supplementary material for: Sex differences in coronary angiographic findings in patients with stable chest pain: analysis of data from the KoRean wOmen’S chest pain rEgistry (KoROSE)
Source: Biol Sex Differ. 2022 Jan 3;13:2. doi: 10.1186/s13293-021-00411-1 (PMC8722299; doi:10.1186/s13293-021-00411-1)
Supplement: Supplementary file 1 — Additional file 1. Table S1. Multiple binary logistic regression analyses showing independent predictors for LM disease and three vessel disease. [file 13293_2021_411_MOESM1_ESM.docx]

**Additional Table S1. Multiple binary logistic regression analyses showing independent predictors for LM disease and three vessel disease**

| **Variable** | **OR (95% CI)** | ***P* value** |
| --- | --- | --- |
| *LM disease (≥ 50%)* |  |  |
| Age ≥ 65 years | 2.34 (1.20-4.59) | 0.013 |
| Men (*vs.* women) | 7.46 (3.48-15.97) | < 0.001 |
| Body mass index ≥ 25 kg/m^2^ | 0.56 (0.29-1.09) | 0.091 |
| Hypertension | 1.28 (0.64-2.54) | 0.474 |
| Diabetes mellitus | 1.38 (0.68-2.81) | 0.369 |
| Dyslipidemia | 0.89 (0.41-1.90) | 0.751 |
| Cigarette smoking | 0.88 (0.40-1.89) | 0.742 |
| GFR < 60 mL/min/1.73m^2^ | 0.77 (0.29-2.07) | 0.611 |
| *Three-vessel disease* |  |  |
| Age ≥ 65 years | 2.50 (1.45-4.32) | 0.001 |
| Men (*vs.* women) | 2.70 (1.57-4.64) | < 0.001 |
| Body mass index ≥ 25 kg/m^2^ | 1.24 (0.75-2.05) | 0.394 |
| Hypertension | 1.81 (1.01-3.26) | 0.048 |
| Diabetes mellitus | 2.78 (1.68-4.60) | < 0.001 |
| Dyslipidemia | 1.43(0.85-2.42) | 0.173 |
| Cigarette smoking | 1.32 (0.70-2.50) | 0.381 |
| GFR < 60 mL/min/1.73m^2^ | 1.39 (0.77-2.51) | 0.269 |

CAD, coronary artery disease; OR, odds ratio; CI, confidence interval; LM, left main; GFR, glomerular filtration rate.
